# (2,2′-Bi­pyridine)­chlorido­[diethyl (2,2′:6′,2′′-terpyridin-4-yl)phospho­nate]ruthenium(II) hexa­fluorido­phosphate aceto­nitrile/water solvate

**DOI:** 10.1107/S1600536813022940

**Published:** 2013-08-21

**Authors:** Weizhong Chen, Francisca N. Rein, Brian L. Scott, Reginaldo C. Rocha

**Affiliations:** a Los Alamos National Laboratory, Los Alamos, NM 87545, USA

## Abstract

The cationic complex in the title compound, [RuCl(C_10_H_8_N_2_)(C_19_H_20_N_3_O_3_P)]PF_6_·0.83CH_3_CN·0.17H_2_O, is a water-oxidation precatalyst functionalized for TiO_2_ attachment *via* terpyridine phospho­nate. The The Ru^II^ atom in the complex has a distorted octa­hedral geometry due to the restricted bite angle [159.50 (18)°] of the terpyridyl ligand. The dihedral angle between the least-squares planes of the terpyridyl and bipyridyl moieties is 86.04 (14)°. The mean Ru—N bond length for bi­pyridine is 2.064 (5) Å, with the bond opposite to Ru—Cl being 0.068 Å shorter. For the substituted terpyridine, the mean Ru—N distance involving the outer N atoms *trans* to each other is 2.057 (6) Å, whereas the bond length involving the central N atom is 1.944 (5) Å. The Ru—Cl distance is 2.4073 (15) Å. The P atom of the phospho­nate group lies in the same plane as its adjacent pyridyl ring, with the ordinary character of the bond between P and C_tpy_ [1.801 (6) Å] allowing for free rotation of the terpyridine substituent around the P—C_tpy_ axis. The aceto­nitrile solvent mol­ecule was refined to be disordered with two water mol­ecules; occupancies for the acetontrile and water mol­ecules were 0.831 (9) and 0.169 (9), respectively. Also disordered was the PF_6_^−^ counter-ion (over three positions) and one of the eth­oxy substituents (with two positions). The crystal structure shows significant intra- and inter­molecular H⋯*X* contacts, especially some involving the Cl^−^ ligand.

## Related literature

For a related crystal structure, see: Zakeeruddin *et al.* (1997 ▶). For the structures of terpyrid­yl/bipyridyl Ru^II^-chlorido compounds relevant to the comparative discussion, see: Chen *et al.* (2011 ▶, 2013 ▶); Jude *et al.* (2008 ▶, 2009 ▶, 2013 ▶). For literature used in the synthetic preparations, see: Evans *et al.* (1973 ▶); Jakubikova *et al.* (2009 ▶); Zakeeruddin *et al.* (1997 ▶). For the catalytic properties of related complexes, see: Chen *et al.* (2009 ▶); Concepcion *et al.* (2008 ▶); Masaoka & Sakai (2009 ▶); Tseng *et al.* (2008 ▶); Wasylenko *et al.* (2010 ▶); Yagi *et al.* (2011 ▶). 
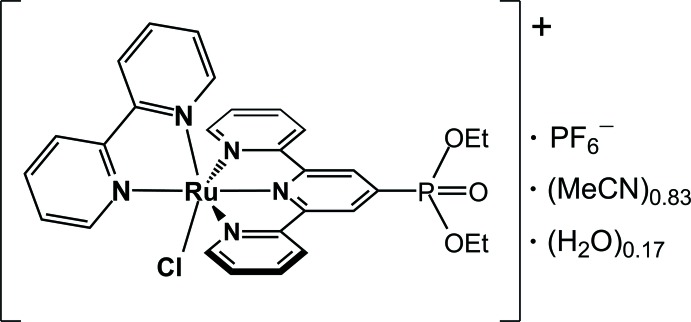


## Experimental

### 

#### Crystal data

[RuCl(C_10_H_8_N_2_)(C_19_H_20_N_3_O_3_P)]PF_6_·0.83C_2_H_3_N·0.17H_2_O
*M* *_r_* = 847.23
Monoclinic, 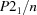

*a* = 8.6367 (14) Å
*b* = 31.515 (5) Å
*c* = 12.696 (2) Å
β = 100.155 (2)°
*V* = 3401.5 (9) Å^3^
*Z* = 4
Mo *K*α radiation
μ = 0.71 mm^−1^
*T* = 120 K
0.28 × 0.20 × 0.08 mm

#### Data collection

Bruker D8 with APEXII CCD diffractometer
Absorption correction: multi-scan (*SADABS*; Bruker, 2007 ▶) *T*_min_ = 0.826, *T*_max_ = 0.945
24414 measured reflections
6233 independent reflections
5056 reflections with *I* > 2σ(*I*)
*R*_int_ = 0.055

#### Refinement

*R*[*F* ^2^ > 2σ(*F* ^2^)] = 0.062
*wR*(*F* ^2^) = 0.141
*S* = 1.14
6233 reflections
574 parameters
394 restraints
H atoms treated by a mixture of independent and constrained refinement
Δρ_max_ = 0.94 e Å^−3^
Δρ_min_ = −1.20 e Å^−3^

### 

Data collection: *APEX2* (Bruker, 2007 ▶); cell refinement: *SAINT-Plus* (Bruker, 2007 ▶); data reduction: *SAINT-Plus*; program(s) used to solve structure: *SHELXS97* (Sheldrick, 2008 ▶); program(s) used to refine structure: *SHELXL2013* (Sheldrick, 2008 ▶) and *SHELXLE* (Hübschle *et al.*, 2011 ▶); molecular graphics: *SHELXTL* (Sheldrick, 2008 ▶); software used to prepare material for publication: *publCIF* (Westrip, 2010 ▶).

## Supplementary Material

Crystal structure: contains datablock(s) I, New_Global_Publ_Block. DOI: 10.1107/S1600536813022940/zl2555sup1.cif

Structure factors: contains datablock(s) I. DOI: 10.1107/S1600536813022940/zl2555Isup2.hkl

Additional supplementary materials:  crystallographic information; 3D view; checkCIF report

## Figures and Tables

**Table 1:** Selected bond lengths (Å)

Ru1—N2	1.944 (5)
Ru1—N4	2.030 (5)
Ru1—N1	2.053 (5)
Ru1—N3	2.061 (5)
Ru1—N5	2.098 (5)
Ru1—Cl1	2.4073 (15)
P1—O1	1.483 (5)
P1—O2	1.540 (5)
P1—O3*B*	1.541 (6)
P1—O3	1.554 (17)
P1—C8	1.801 (6)

**Fig. 1.**
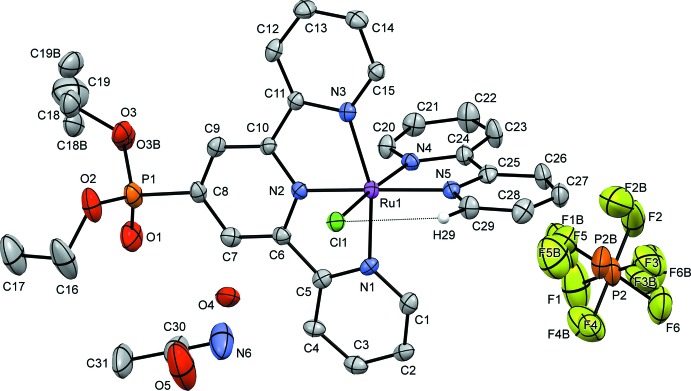
Single crystal structure of [Ru(Cl)(bpy)(tpy-p)](PF6)×MeCN. Displacement ellipsoids are drawn at the 50% probability level. Except for H29 (which is involved in the intramolecular H···X contact with the Cl- ligand), hydrogen atoms are omitted for clarity.

**Fig. 2.**
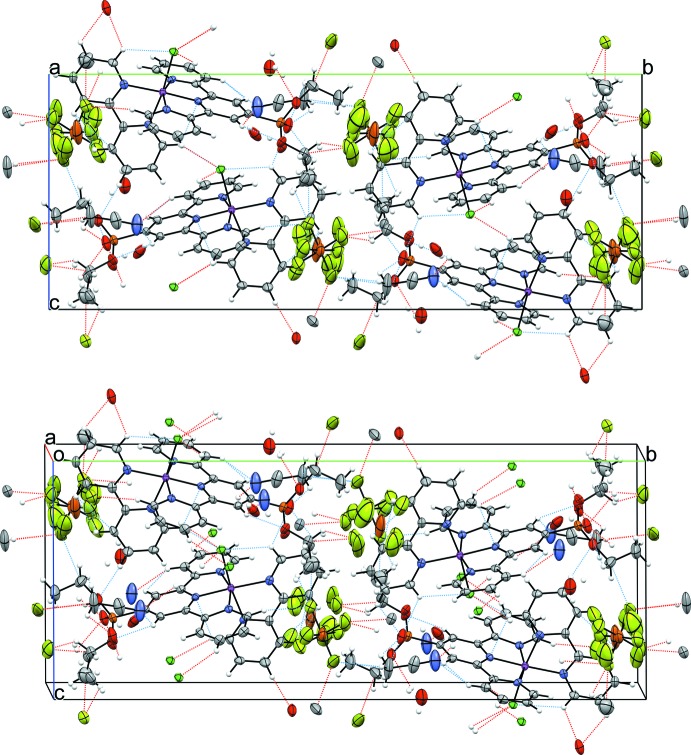
Two views of the crystal packing diagram of [Ru(Cl)(bpy)(tpy-p)](PF6)×MeCN. Nonbonded short contacts are indicated by cyan dotted lines (expanded contacts) and red dotted lines (hanging contacts). For clarity, only contacts that are structurally relevant and at least 0.1 Å shorter than the sum of van der Waals radii are shown.

## supplementary crystallographic information

### 1. Comment

A crucial challenge to renewable energy technologies based on artificial photosynthesis and production of solar fuels has been the development of efficient catalysts for splitting water, with evolution of H_2_ and O_2_. The complete four-electron oxidation of water into dioxygen, in particular, is a semi-reaction of tremendous complexity. Recently, mononuclear ruthenium complexes such as [Ru^II^(OH_2_)(bpy)(tpy)]^2+^ (bpy = 2,2'-bipyridine; tpy = 2,2':6',2"-terpyridine) and its structural analogues have emerged as catalysts for water oxidation (for example, see: Concepcion *et al.*, 2008; Masaoka & Sakai, 2009; Tseng *et al.*, 2008; Wasylenko *et al.*, 2010; Yagi *et al.*, 2011). In these systems, the catalytic aquo species is readily prepared in water by ligand substitution at the chloro precursor/precatalyst, [Ru^II^(Cl)(bpy)(tpy)]^+^ (Jakubikova *et al.*, 2009). In order to heterogenize this precatalyst by attachment onto TiO_2_ surfaces, we have synthesized the title complex [Ru^II^(Cl)(bpy)(tpy-p)]^+^ (I; tpy-p = diethyl 2,2':6',2"-terpyridine-4'-phosphonate). The phosphonate group in its diethyl ester form can then be hydrolized in acidic medium to yield its phosphonic acid, which is well known as an efficient TiO_2_ anchoring group upon deprotonation. This approach has also been well demonstrated for related complexes as photosensitizers in dye-sensitized solar cells (Zakeeruddin *et al.*, 1997). Despite the relevance of such phosphonated terpyridyl Ru complexes to these energy-related research areas, crystallographically characterized structures containing the tpy-PO_3_ ligand moiety are still scarce (Zakeeruddin *et al.*, 1997).

The hexafluorophosphate salt of I crystallized in the monoclinic space group (*P*2_1_/*n*) from an acetonitrile solution. Its crystal structure is shown in Figs. 1 and 2. The cationic complex has a distorted octahedral geometry due to the restricted bite angle of the meridionally coordinated tridendate terpyridyl ligand. The N1—Ru—N3 angle of 159.50 (18)° is very similar to those recently reported for bis-terpyridyl Ru(II) complexes (Chen *et al.*, 2013; Jude *et al.*, 2013), and far from the ideal angle of 180° in an octahedral geometry. The bpy ligand has a *cis* configuration, with the N4—Ru—N5 angle of 78.45 (19)° consistent with those typically found in Ru^II^-bpy complexes (Chen *et al.*, 2011; Jude *et al.*, 2008). The bpy-N4 atom is arranged *trans* to the chloride ligand in a nearly linear N—Ru—Cl fashion (172.62 (14)°). The Ru center and atoms N2, N4, N5, and Cl1 form an equatorial plane with a maximum deviation of 0.031 (4) Å from ideal planarity (N5). The bipyridyl and terpyridyl moieties are approximately planar (with maximum deviations of 0.087 (6) Å and 0.146 (6) Å, respectively) and their mean planes are essentially perpendicular to each other with a dihedral angle of 86.04 (14)°. Although Ru is practically coplanar with the bpy plane (deviation of 0.002 (1) Å), it deviates significantly from the tpy plane (0.143 (1) Å).

For the tpy-p ligand, the mean Ru—N distance involving the outer nitrogen atoms *trans* to each other is 2.057 (5) Å whereas the bond distance involving the central nitrogen is much shorter (1.944 (5) Å), as a result of the structural constraint imposed by these *mer*-arranged tridendate ligands (Chen *et al.*, 2013; Jude *et al.*, 2013). For the bpy ligand, the Ru—N bond distance is 2.098 (5) Å for N5 but only 2.030 (5) for N4, reflecting the increased Ru^II^→N_bpy_π-backbonding interaction at the coordinating atom *trans* to the π-donor Cl^-^ ligand. The Ru—Cl distance of 2.4073 (15) Å is nearly the same as those observed in related structures (Jude *et al.*, 2009). An *intra*molecular H···Cl contact of 2.71 Å exists between Cl1 and the hydrogen atom of the nearest C atom (H29), similar to our previous observations (Chen *et al.*, 2011; Jude *et al.*, 2009). Significant *inter*molecular contacts of 2.76 Å, 2.81 Å, and 2.85 Å betwen Cl and H3, H13, and H20 are also found, but these are closer to the sum of the van der Waals radii for hydrogen and chlorine (2.95 Å).

The P atom of the anchoring phosphonate substituent lies in the same plane as its adjacent pyridyl ring, with a maximum deviation of 0.023 (2) Å from coplanarity. The length of the formally P═O bond between P1 and O1 (1.483 (5) Å) is only about 0.06 Å shorter than that of P—O(Et) involving P1 and O2(C16H_2_C17H_3_) and O3(C18H_2_C19H_3_). That is partly attributed to the multiple intermolecular interactions involving these O atoms. The bond lengths and angles involving the P and O atoms are compiled along with the selected data in Table 1. The observed P1—C8 bond length of 1.801 (6) Å is typical of ordinary P—C(aromatic) bonds. As pointed earlier (Zakeeruddin *et al.*, 1997), this ordinary character of the P—C bond allows for free rotation of the phosphonate group around the P—C_tpy_ axis.

The acetonitrile solvate molecule was refined to be disordered with two water molecules; occupancies for the acetontrile and water molecules were 0.831 (9) and 0.169 (9), respectively. One of the ethoxy substituents (O3(C18H_2_C19H_3_)) was refined as disordered with two moieties; occupancies were 0.793 (13) and 0.207 (13). Also disordered was the PF_6_^-^ counterion, which was refined over two different moieties (Figs. 1 and 2), occupancies refined to 0.726 (14) and 0.274 (14). Although classic H bonds are not found in the crystal structure of I(PF_6_)×MeCN, several intermolecular contacts (*i.e.*, distances shorter than the sum of van der Waals radii) exist between cations (I) as well as between the cation and its counterion (PF_6_^-^) or solvate molecules. Those that appear to be more relevant to the crystal-packing driving forces are explicitly shown in Fig. 2.

The identity of the cation [Ru(Cl)(bpy)(tpy-p)]^+^ (I) was also characterized in MeCN solutions by several techniques. Mass spectra (ESI-MS: *m/z* 660.3) are in agreement with the formulation as [(M—PF_6_^-^)^+^] for the cation I (calcd for C_29_H_28_ClN_5_O_3_PRu, *m/z* 662.1). Electrochemical measurements by cyclic voltammetry gave a redox potential of 0.88 V *versus* SCE for the reversible Ru^II^/Ru^III^ couple. This potential is positively shifted by 70 mV relative to the unmodified [Ru(Cl)(bpy)(tpy)]^+^ complex (0.81 V *versus* SCE; Chen *et al.*, 2009), which is consistent with the electron-withdrawing nature of the phosphonate substituent in tpy-p. Upon surface tethering, this is a desirable feature because it facilitates pulling the metal→ligand charge toward the functionalized tpy ligand for injection into the conduction band of TiO_2_.

### 2. Experimental

The synthesis of [Ru(Cl)(bpy)(tpy-p)]PF_6_ was performed stepwise through a procedure involving the intermediate RuCl_2_(DMSO)(tpy-p). First, this intermediate was obtained by reacting stoichiometric amounts (1.0 mmol) of RuCl_2_(DMSO)_4_ (Evans *et al.*, 1973) with diethyl 2,2':6',2"-terpyridine-4'-phosphonate (Zakeeruddin *et al.*, 1997) in 75 ml of dry EtOH/MeOH (4:1) heated at reflux for ~4 h, under an Ar atmosphere. To the intermediate product was then added 2,2'-bipyridine (20% excess) and the next step also proceeded for ~4 h, under the same conditions. The reaction solution was cooled down to room temperature and excess NH_4_PF_6_ was added to form the red precipitate, which was collected by filtration and then rinsed with Et_2_O and dried under vacuum. Further purification was performed by column chromatography. The overall yield was relatively low (30%). When the same reaction was carried out in the presence of water (EtOH/H_2_O, 2:1), the partially hydrolized product (*i.e.* [Ru(Cl)(bpy)(tpy-P(O)(OH)(OEt))]PF_6_ could be obtained in much higher yields (60%), but this product was not characterized by X-ray crystallography. For the structure of [Ru(Cl)(bpy)(tpy-p)]PF_6_ reported herein, single crystals suitable for X-ray analysis were grown by slow diffusion of Et_2_O into MeCN solutions of the complex in a long thin tube.

### 3. Refinement

All carbon-bound hydrogen atom positions were idealized, and were set to ride on the atom they were attached to. An acetonitrile solvate molecule was refined to be disordered with two water molecules. C, N and O atoms of these solvate molecules were refined anisotropically without application of restraints or constraints. Water H atoms were restrained to have O—H bonding distances of 0.82 (2) Å, and intramolecular H···H distances of 1.36 Å. Occupancies for the acetontrile and water molecules refined to 0.831 (9) and 0.169 (9), respectively. One of the ethoxy substituents was refined as disordered with two moieties. Bond distances were restrained to be the same as for the not disordered ethoxy group (esd = 0.02 Å), and the P—O distances within the two disordered moieties was restrained to be the same (esd = 0.02 Å). The two oxygen atoms were constrained to have identical ADPs, the Uij components of neighboring disordered atoms were restrained to be similar (esd = 0.01 Å^2^), and the ADPs of the methyl C atoms were restrained to be approximately isotropic (esd 0.01 Å^2^). Occupancies refined to 0.793 (13) and 0.207 (13), respectively. The PF_6_ anion was refined as disordered over two different moieties. All P—F bond distances were restrained to be similar (esd 0.02 Å), as were all intramolecular F···F distances of directly neighboring fluorine atoms. Uij components of P and F atoms were restrained to be similar, as were the components of the ADPs in the direction of the bonds (SIMU and DELU restraints in SHELXL, esd = 0.01 Å^2^ for both). Occupancies refined to 0.726 (14) and 0.274 (14). The final refinement included anisotropic temperature factors on all non-hydrogen atoms.

### Crystal data

**Table:**

[RuCl(C_10_H_8_N_2_)(C_19_H_20_N_3_O_3_P)]PF_6_·0.83C_2_H_3_N·0.17H_2_O	*F*(000) = 1710.6
*M**_r_* = 847.23	*D*_x_ = 1.655 Mg m^−^^3^
Monoclinic, *P*2_1_/*n*	Mo *K*α radiation, λ = 0.71073 Å
*a* = 8.6367 (14) Å	Cell parameters from 4889 reflections
*b* = 31.515 (5) Å	θ = 5.0–49.1°
*c* = 12.696 (2) Å	µ = 0.71 mm^−^^1^
β = 100.155 (2)°	*T* = 120 K
*V* = 3401.5 (9) Å^3^	Block, red
*Z* = 4	0.28 × 0.20 × 0.08 mm

### Data collection

**Table:**

Bruker D8 with APEXII CCD diffractometer	6233 independent reflections
Radiation source: fine-focus sealed tube	5056 reflections with *I* > 2σ(*I*)
Graphite monochromator	*R*_int_ = 0.055
ω scans	θ_max_ = 25.4°, θ_min_ = 2.1°
Absorption correction: multi-scan (*SADABS*; Bruker, 2007)	*h* = −10→10
*T*_min_ = 0.826, *T*_max_ = 0.945	*k* = −37→38
24414 measured reflections	*l* = −15→15

### Refinement

**Table:**

Refinement on *F*^2^	Primary atom site location: structure-invariant direct methods
Least-squares matrix: full	Secondary atom site location: difference Fourier map
*R*[*F*^2^ > 2σ(*F*^2^)] = 0.062	Hydrogen site location: mixed
*wR*(*F*^2^) = 0.141	H atoms treated by a mixture of independent and constrained refinement
*S* = 1.14	*w* = 1/[σ^2^(*F*_o_^2^) + (0.0274*P*)^2^ + 23.9905*P*] where *P* = (*F*_o_^2^ + 2*F*_c_^2^)/3
6233 reflections	(Δ/σ)_max_ = 0.003
574 parameters	Δρ_max_ = 0.94 e Å^−^^3^
394 restraints	Δρ_min_ = −1.20 e Å^−^^3^

### Special details

**Table:**

Geometry. All esds (except the esd in the dihedral angle between two l.s. planes) are estimated using the full covariance matrix. The cell esds are taken into account individually in the estimation of esds in distances, angles and torsion angles; correlations between esds in cell parameters are only used when they are defined by crystal symmetry. An approximate (isotropic) treatment of cell esds is used for estimating esds involving l.s. planes.

### Fractional atomic coordinates and isotropic or equivalent isotropic displacement parameters (Å^2^)

**Table:**

	*x*	*y*	*z*	*U*_iso_*/*U*_eq_	Occ. (<1)
Ru1	0.11155 (5)	0.19185 (2)	0.07673 (4)	0.01776 (14)	
Cl1	−0.03875 (17)	0.21030 (4)	−0.09533 (11)	0.0228 (3)	
P1	0.1827 (2)	0.39059 (5)	0.20621 (14)	0.0343 (4)	
O1	0.3502 (6)	0.40325 (15)	0.2183 (5)	0.0492 (14)	
N1	0.3155 (5)	0.20702 (15)	0.0232 (4)	0.0188 (10)	
N2	0.1283 (5)	0.25162 (14)	0.1148 (4)	0.0180 (10)	
N3	−0.0819 (5)	0.19850 (14)	0.1500 (4)	0.0200 (10)	
N4	0.2321 (6)	0.16840 (15)	0.2163 (4)	0.0205 (11)	
N5	0.1106 (5)	0.12661 (15)	0.0439 (4)	0.0208 (11)	
C1	0.4122 (7)	0.18137 (19)	−0.0200 (5)	0.0251 (14)	
H1	0.3820	0.1526	−0.0336	0.030*	
C2	0.5521 (7)	0.1947 (2)	−0.0452 (5)	0.0243 (13)	
H2	0.6182	0.1753	−0.0737	0.029*	
C3	0.5956 (7)	0.2366 (2)	−0.0285 (5)	0.0286 (14)	
H3	0.6914	0.2465	−0.0465	0.034*	
C4	0.4985 (7)	0.2640 (2)	0.0146 (5)	0.0249 (13)	
H4	0.5263	0.2930	0.0260	0.030*	
C5	0.3599 (7)	0.24847 (18)	0.0409 (5)	0.0208 (12)	
C6	0.2522 (7)	0.27427 (18)	0.0923 (5)	0.0215 (13)	
C7	0.2689 (7)	0.31671 (19)	0.1202 (5)	0.0249 (13)	
H7	0.3569	0.3325	0.1065	0.030*	
C8	0.1540 (7)	0.33567 (18)	0.1686 (5)	0.0238 (13)	
C9	0.0274 (7)	0.31249 (18)	0.1888 (5)	0.0235 (13)	
H9	−0.0523	0.3257	0.2201	0.028*	
C10	0.0168 (7)	0.26958 (18)	0.1630 (4)	0.0196 (12)	
C11	−0.1020 (7)	0.23929 (17)	0.1854 (4)	0.0194 (12)	
C12	−0.2213 (7)	0.24910 (19)	0.2401 (5)	0.0257 (14)	
H12	−0.2356	0.2775	0.2613	0.031*	
C13	−0.3200 (7)	0.2175 (2)	0.2640 (5)	0.0296 (15)	
H13	−0.4013	0.2239	0.3031	0.036*	
C14	−0.2997 (7)	0.1765 (2)	0.2308 (5)	0.0284 (14)	
H14	−0.3662	0.1543	0.2468	0.034*	
C15	−0.1808 (7)	0.16847 (18)	0.1738 (5)	0.0220 (13)	
H15	−0.1682	0.1403	0.1502	0.026*	
O2	0.0733 (6)	0.41696 (14)	0.1220 (4)	0.0476 (14)	
C16	0.1522 (13)	0.4448 (3)	0.0503 (7)	0.073 (3)	
H16A	0.2530	0.4319	0.0401	0.088*	
H16B	0.0839	0.4476	−0.0207	0.088*	
C17	0.1817 (14)	0.4879 (2)	0.1013 (8)	0.077 (3)	
H17A	0.2051	0.5083	0.0480	0.116*	
H17B	0.2712	0.4863	0.1605	0.116*	
H17C	0.0880	0.4972	0.1287	0.116*	
O3	0.068 (3)	0.3923 (10)	0.288 (2)	0.0376 (17)	0.207 (13)
C18	0.000 (4)	0.4298 (10)	0.338 (3)	0.043 (3)	0.207 (13)
H18A	−0.0050	0.4550	0.2911	0.051*	0.207 (13)
H18B	−0.1066	0.4232	0.3514	0.051*	0.207 (13)
C19	0.111 (6)	0.4375 (16)	0.442 (3)	0.079 (17)	0.207 (13)
H19A	0.2185	0.4403	0.4279	0.119*	0.207 (13)
H19B	0.1057	0.4135	0.4902	0.119*	0.207 (13)
H19C	0.0805	0.4636	0.4747	0.119*	0.207 (13)
O3B	0.1198 (10)	0.3933 (2)	0.3123 (5)	0.0376 (17)	0.793 (13)
C18B	0.1154 (12)	0.4349 (3)	0.3644 (8)	0.040 (2)	0.793 (13)
H18C	0.0701	0.4565	0.3112	0.048*	0.793 (13)
H18D	0.2232	0.4438	0.3968	0.048*	0.793 (13)
C19B	0.0162 (14)	0.4307 (3)	0.4489 (8)	0.050 (3)	0.793 (13)
H19D	0.0083	0.4583	0.4831	0.075*	0.793 (13)
H19E	0.0643	0.4101	0.5027	0.075*	0.793 (13)
H19F	−0.0891	0.4209	0.4164	0.075*	0.793 (13)
C20	0.2890 (7)	0.19166 (19)	0.3034 (5)	0.0260 (13)	
H20	0.2796	0.2217	0.2986	0.031*	
C21	0.3592 (8)	0.1744 (2)	0.3978 (5)	0.0298 (15)	
H21	0.3961	0.1921	0.4575	0.036*	
C22	0.3761 (9)	0.1312 (2)	0.4060 (6)	0.0406 (18)	
H22	0.4244	0.1185	0.4714	0.049*	
C23	0.3218 (9)	0.1067 (2)	0.3179 (5)	0.0382 (17)	
H23	0.3341	0.0768	0.3217	0.046*	
C24	0.2493 (7)	0.12545 (18)	0.2234 (5)	0.0253 (14)	
C25	0.1897 (7)	0.10225 (18)	0.1243 (5)	0.0245 (13)	
C26	0.2106 (8)	0.05900 (19)	0.1113 (5)	0.0324 (15)	
H26	0.2660	0.0423	0.1680	0.039*	
C27	0.1489 (8)	0.0409 (2)	0.0138 (5)	0.0320 (15)	
H27	0.1618	0.0113	0.0029	0.038*	
C28	0.0690 (7)	0.0655 (2)	−0.0675 (5)	0.0317 (15)	
H28	0.0270	0.0533	−0.1350	0.038*	
C29	0.0510 (7)	0.10805 (19)	−0.0493 (5)	0.0270 (14)	
H29	−0.0059	0.1250	−0.1050	0.032*	
P2	0.7996 (7)	0.04256 (16)	0.2491 (4)	0.0514 (14)	0.726 (14)
F1	0.9091 (15)	0.0792 (2)	0.3042 (7)	0.102 (4)	0.726 (14)
F2	0.7768 (19)	0.0239 (2)	0.3607 (7)	0.124 (5)	0.726 (14)
F3	0.6949 (13)	0.0053 (3)	0.1890 (9)	0.092 (3)	0.726 (14)
F4	0.8227 (9)	0.0618 (2)	0.1346 (5)	0.048 (2)	0.726 (14)
F5	0.6492 (13)	0.0715 (3)	0.2426 (9)	0.092 (3)	0.726 (14)
F6	0.9482 (10)	0.0134 (2)	0.2475 (7)	0.083 (3)	0.726 (14)
P2B	0.755 (2)	0.0482 (6)	0.2461 (15)	0.109 (6)	0.274 (14)
F1B	0.800 (4)	0.0835 (7)	0.3349 (18)	0.118 (8)	0.274 (14)
F2B	0.637 (4)	0.0278 (7)	0.313 (2)	0.130 (9)	0.274 (14)
F3B	0.712 (4)	0.0119 (8)	0.159 (2)	0.136 (9)	0.274 (14)
F4B	0.872 (3)	0.0681 (8)	0.177 (3)	0.134 (10)	0.274 (14)
F5B	0.620 (3)	0.0777 (8)	0.185 (2)	0.110 (8)	0.274 (14)
F6B	0.895 (3)	0.0194 (8)	0.307 (3)	0.181 (10)	0.274 (14)
N6	0.6748 (10)	0.3516 (3)	0.1400 (10)	0.072 (3)	0.831 (9)
C30	0.6579 (11)	0.3871 (3)	0.1224 (10)	0.056 (3)	0.831 (9)
C31	0.6410 (14)	0.4298 (3)	0.1053 (12)	0.081 (4)	0.831 (9)
H31A	0.5756	0.4417	0.1537	0.121*	0.831 (9)
H31B	0.5906	0.4350	0.0310	0.121*	0.831 (9)
H31C	0.7447	0.4434	0.1188	0.121*	0.831 (9)
O4	0.680 (3)	0.3472 (7)	0.254 (2)	0.055 (11)	0.169 (9)
H4A	0.679 (6)	0.3723 (8)	0.271 (6)	0.066*	0.169 (9)
H4B	0.592 (4)	0.3385 (13)	0.228 (7)	0.066*	0.169 (9)
O5	0.505 (5)	0.3723 (8)	−0.026 (2)	0.101 (19)	0.169 (9)
H5A	0.462 (8)	0.3767 (17)	−0.088 (2)	0.122*	0.169 (9)
H5B	0.541 (9)	0.3941 (10)	0.004 (3)	0.122*	0.169 (9)

### Atomic displacement parameters (Å^2^)

**Table:**

	*U*^11^	*U*^22^	*U*^33^	*U*^12^	*U*^13^	*U*^23^
Ru1	0.0167 (2)	0.0161 (2)	0.0199 (2)	0.00053 (19)	0.00165 (17)	−0.00016 (19)
Cl1	0.0219 (7)	0.0231 (7)	0.0227 (7)	0.0017 (6)	0.0021 (6)	0.0014 (6)
P1	0.0517 (12)	0.0200 (8)	0.0308 (10)	−0.0051 (8)	0.0062 (8)	−0.0059 (7)
O1	0.050 (3)	0.027 (3)	0.070 (4)	−0.012 (2)	0.010 (3)	−0.014 (2)
N1	0.017 (2)	0.023 (2)	0.016 (2)	0.0008 (19)	0.001 (2)	−0.004 (2)
N2	0.018 (2)	0.019 (2)	0.017 (2)	0.0009 (19)	0.001 (2)	0.0020 (19)
N3	0.019 (2)	0.020 (2)	0.020 (2)	0.001 (2)	0.000 (2)	0.000 (2)
N4	0.020 (3)	0.022 (3)	0.020 (3)	0.001 (2)	0.003 (2)	0.004 (2)
N5	0.017 (2)	0.020 (2)	0.026 (3)	0.0003 (19)	0.003 (2)	0.002 (2)
C1	0.024 (3)	0.027 (3)	0.022 (3)	0.005 (3)	−0.001 (3)	0.000 (3)
C2	0.020 (3)	0.033 (3)	0.019 (3)	0.006 (3)	0.004 (2)	0.004 (3)
C3	0.022 (3)	0.037 (4)	0.026 (3)	−0.002 (3)	0.004 (3)	0.006 (3)
C4	0.020 (3)	0.030 (3)	0.024 (3)	−0.004 (3)	0.004 (3)	0.001 (3)
C5	0.017 (3)	0.026 (3)	0.018 (3)	0.002 (2)	−0.001 (2)	0.000 (2)
C6	0.022 (3)	0.023 (3)	0.020 (3)	0.001 (2)	0.003 (2)	0.003 (2)
C7	0.025 (3)	0.027 (3)	0.023 (3)	−0.004 (3)	0.004 (3)	−0.002 (3)
C8	0.033 (3)	0.018 (3)	0.018 (3)	−0.001 (3)	0.001 (3)	−0.001 (2)
C9	0.027 (3)	0.024 (3)	0.018 (3)	0.004 (3)	0.001 (2)	−0.002 (2)
C10	0.017 (3)	0.022 (3)	0.019 (3)	0.006 (2)	0.003 (2)	0.004 (2)
C11	0.019 (3)	0.020 (3)	0.018 (3)	0.002 (2)	0.001 (2)	0.001 (2)
C12	0.024 (3)	0.024 (3)	0.027 (3)	0.006 (3)	−0.001 (3)	−0.004 (3)
C13	0.022 (3)	0.034 (4)	0.034 (4)	−0.001 (3)	0.009 (3)	0.001 (3)
C14	0.019 (3)	0.034 (3)	0.032 (4)	0.000 (3)	0.004 (3)	0.008 (3)
C15	0.020 (3)	0.022 (3)	0.023 (3)	0.002 (2)	0.002 (3)	0.002 (2)
O2	0.067 (4)	0.022 (2)	0.046 (3)	−0.004 (2)	−0.009 (3)	0.000 (2)
C16	0.128 (9)	0.042 (5)	0.041 (5)	−0.012 (5)	−0.011 (5)	0.016 (4)
C17	0.117 (9)	0.032 (4)	0.078 (7)	−0.020 (5)	0.003 (6)	−0.004 (4)
O3	0.055 (4)	0.026 (2)	0.033 (3)	0.002 (3)	0.010 (3)	−0.011 (3)
C18	0.058 (7)	0.028 (6)	0.041 (6)	−0.001 (6)	0.009 (6)	−0.007 (6)
C19	0.08 (2)	0.08 (2)	0.08 (2)	−0.021 (18)	0.018 (18)	−0.008 (18)
O3B	0.055 (4)	0.026 (2)	0.033 (3)	0.002 (3)	0.010 (3)	−0.011 (3)
C18B	0.056 (5)	0.024 (4)	0.042 (4)	−0.008 (4)	0.016 (4)	−0.011 (3)
C19B	0.060 (7)	0.038 (5)	0.056 (6)	−0.006 (5)	0.024 (5)	−0.020 (5)
C20	0.025 (3)	0.022 (3)	0.029 (3)	0.003 (3)	−0.002 (3)	−0.002 (3)
C21	0.032 (4)	0.033 (3)	0.021 (3)	0.001 (3)	−0.003 (3)	−0.002 (3)
C22	0.051 (5)	0.034 (4)	0.030 (4)	0.005 (3)	−0.010 (3)	−0.001 (3)
C23	0.055 (5)	0.022 (3)	0.034 (4)	0.008 (3)	−0.002 (3)	0.009 (3)
C24	0.027 (3)	0.018 (3)	0.031 (4)	0.000 (2)	0.006 (3)	0.000 (3)
C25	0.015 (3)	0.024 (3)	0.034 (4)	0.001 (2)	0.005 (3)	0.001 (3)
C26	0.042 (4)	0.018 (3)	0.036 (4)	0.003 (3)	0.002 (3)	0.005 (3)
C27	0.040 (4)	0.019 (3)	0.037 (4)	0.003 (3)	0.007 (3)	0.000 (3)
C28	0.026 (3)	0.032 (4)	0.035 (4)	−0.002 (3)	−0.001 (3)	−0.010 (3)
C29	0.026 (3)	0.029 (3)	0.024 (3)	0.003 (3)	−0.003 (3)	0.002 (3)
P2	0.078 (3)	0.023 (2)	0.057 (2)	0.0126 (18)	0.022 (2)	0.0061 (15)
F1	0.167 (9)	0.049 (4)	0.071 (6)	0.001 (5)	−0.027 (6)	−0.013 (4)
F2	0.274 (15)	0.048 (4)	0.075 (5)	0.056 (7)	0.102 (7)	0.021 (4)
F3	0.134 (7)	0.044 (4)	0.115 (7)	−0.035 (5)	0.067 (6)	−0.008 (5)
F4	0.058 (4)	0.037 (4)	0.051 (4)	−0.003 (3)	0.011 (3)	0.008 (3)
F5	0.115 (7)	0.067 (5)	0.111 (9)	0.039 (5)	0.068 (6)	0.021 (5)
F6	0.121 (6)	0.051 (4)	0.081 (6)	0.038 (4)	0.028 (5)	0.014 (4)
P2B	0.141 (13)	0.029 (7)	0.187 (13)	0.024 (7)	0.109 (9)	0.014 (6)
F1B	0.16 (2)	0.069 (12)	0.123 (15)	0.005 (13)	0.016 (14)	0.037 (9)
F2B	0.19 (2)	0.071 (15)	0.16 (2)	−0.012 (14)	0.121 (17)	−0.003 (13)
F3B	0.22 (2)	0.050 (12)	0.184 (18)	−0.037 (12)	0.161 (15)	−0.002 (12)
F4B	0.148 (18)	0.079 (17)	0.20 (2)	−0.031 (14)	0.093 (18)	0.013 (16)
F5B	0.124 (15)	0.061 (13)	0.14 (2)	−0.022 (11)	0.025 (14)	−0.010 (13)
F6B	0.199 (19)	0.119 (18)	0.25 (2)	0.077 (17)	0.098 (18)	0.061 (17)
N6	0.040 (5)	0.039 (5)	0.137 (11)	0.003 (4)	0.014 (6)	0.004 (6)
C30	0.041 (6)	0.039 (6)	0.091 (9)	−0.003 (4)	0.021 (5)	−0.014 (5)
C31	0.080 (9)	0.023 (5)	0.150 (13)	−0.007 (5)	0.052 (9)	−0.011 (6)
O4	0.06 (2)	0.05 (2)	0.06 (2)	0.023 (17)	0.039 (19)	0.027 (17)
O5	0.18 (5)	0.04 (2)	0.08 (3)	−0.01 (3)	0.00 (3)	0.00 (2)

### Geometric parameters (Å, º)

**Table:**

Ru1—N2	1.944 (5)	O3—C18	1.509 (17)
Ru1—N4	2.030 (5)	C18—C19	1.502 (18)
Ru1—N1	2.053 (5)	C18—H18A	0.9900
Ru1—N3	2.061 (5)	C18—H18B	0.9900
Ru1—N5	2.098 (5)	C19—H19A	0.9800
Ru1—Cl1	2.4073 (15)	C19—H19B	0.9800
P1—O1	1.483 (5)	C19—H19C	0.9800
P1—O2	1.540 (5)	O3B—C18B	1.472 (9)
P1—O3B	1.541 (6)	C18B—C19B	1.491 (11)
P1—O3	1.554 (17)	C18B—H18C	0.9900
P1—C8	1.801 (6)	C18B—H18D	0.9900
N1—C1	1.347 (8)	C19B—H19D	0.9800
N1—C5	1.369 (7)	C19B—H19E	0.9800
N2—C10	1.353 (7)	C19B—H19F	0.9800
N2—C6	1.358 (7)	C20—C21	1.358 (8)
N3—C15	1.345 (7)	C20—H20	0.9500
N3—C11	1.383 (7)	C21—C22	1.371 (9)
N4—C20	1.345 (7)	C21—H21	0.9500
N4—C24	1.363 (7)	C22—C23	1.372 (9)
N5—C29	1.340 (8)	C22—H22	0.9500
N5—C25	1.361 (7)	C23—C24	1.383 (9)
C1—C2	1.369 (8)	C23—H23	0.9500
C1—H1	0.9500	C24—C25	1.468 (9)
C2—C3	1.378 (9)	C25—C26	1.389 (8)
C2—H2	0.9500	C26—C27	1.382 (9)
C3—C4	1.381 (9)	C26—H26	0.9500
C3—H3	0.9500	C27—C28	1.376 (9)
C4—C5	1.387 (8)	C27—H27	0.9500
C4—H4	0.9500	C28—C29	1.375 (9)
C5—C6	1.472 (8)	C28—H28	0.9500
C6—C7	1.385 (8)	C29—H29	0.9500
C7—C8	1.391 (9)	P2—F1	1.575 (8)
C7—H7	0.9500	P2—F5	1.577 (8)
C8—C9	1.376 (9)	P2—F2	1.578 (8)
C9—C10	1.391 (8)	P2—F6	1.581 (8)
C9—H9	0.9500	P2—F3	1.592 (8)
C10—C11	1.465 (8)	P2—F4	1.620 (7)
C11—C12	1.375 (8)	P2B—F2B	1.577 (16)
C12—C13	1.379 (9)	P2B—F1B	1.582 (16)
C12—H12	0.9500	P2B—F5B	1.583 (16)
C13—C14	1.380 (9)	P2B—F3B	1.586 (17)
C13—H13	0.9500	P2B—F4B	1.586 (16)
C14—C15	1.379 (9)	P2B—F6B	1.597 (16)
C14—H14	0.9500	N6—C30	1.144 (12)
C15—H15	0.9500	C30—C31	1.369 (13)
O2—C16	1.511 (9)	C31—H31A	0.9800
C16—C17	1.507 (10)	C31—H31B	0.9800
C16—H16A	0.9900	C31—H31C	0.9800
C16—H16B	0.9900	O4—H4A	0.8200 (11)
C17—H17A	0.9800	O4—H4B	0.8200 (11)
C17—H17B	0.9800	O5—H5A	0.8200 (11)
C17—H17C	0.9800	O5—H5B	0.8200 (11)
			
N2—Ru1—N4	97.55 (19)	H17B—C17—H17C	109.5
N2—Ru1—N1	79.99 (19)	C18—O3—P1	131 (3)
N4—Ru1—N1	92.05 (19)	C19—C18—O3	105 (2)
N2—Ru1—N3	79.62 (19)	C19—C18—H18A	110.7
N4—Ru1—N3	88.54 (19)	O3—C18—H18A	110.7
N1—Ru1—N3	159.50 (18)	C19—C18—H18B	110.7
N2—Ru1—N5	175.42 (19)	O3—C18—H18B	110.7
N4—Ru1—N5	78.45 (19)	H18A—C18—H18B	108.8
N1—Ru1—N5	97.82 (18)	C18—C19—H19A	109.5
N3—Ru1—N5	102.37 (18)	C18—C19—H19B	109.5
N2—Ru1—Cl1	89.79 (14)	H19A—C19—H19B	109.5
N4—Ru1—Cl1	172.62 (14)	C18—C19—H19C	109.5
N1—Ru1—Cl1	90.03 (13)	H19A—C19—H19C	109.5
N3—Ru1—Cl1	92.00 (13)	H19B—C19—H19C	109.5
N5—Ru1—Cl1	94.25 (13)	C18B—O3B—P1	118.9 (6)
O1—P1—O2	113.2 (3)	O3B—C18B—C19B	107.9 (7)
O1—P1—O3B	112.5 (4)	O3B—C18B—H18C	110.1
O2—P1—O3B	108.0 (4)	C19B—C18B—H18C	110.1
O1—P1—O3	130.2 (12)	O3B—C18B—H18D	110.1
O2—P1—O3	93.4 (13)	C19B—C18B—H18D	110.1
O1—P1—C8	111.8 (3)	H18C—C18B—H18D	108.4
O2—P1—C8	107.2 (3)	C18B—C19B—H19D	109.5
O3B—P1—C8	103.4 (4)	C18B—C19B—H19E	109.5
O3—P1—C8	97.8 (11)	H19D—C19B—H19E	109.5
C1—N1—C5	117.6 (5)	C18B—C19B—H19F	109.5
C1—N1—Ru1	128.7 (4)	H19D—C19B—H19F	109.5
C5—N1—Ru1	113.6 (4)	H19E—C19B—H19F	109.5
C10—N2—C6	121.7 (5)	N4—C20—C21	123.3 (6)
C10—N2—Ru1	119.4 (4)	N4—C20—H20	118.4
C6—N2—Ru1	118.9 (4)	C21—C20—H20	118.4
C15—N3—C11	117.4 (5)	C20—C21—C22	119.4 (6)
C15—N3—Ru1	128.9 (4)	C20—C21—H21	120.3
C11—N3—Ru1	113.5 (4)	C22—C21—H21	120.3
C20—N4—C24	117.8 (5)	C21—C22—C23	118.6 (6)
C20—N4—Ru1	125.2 (4)	C21—C22—H22	120.7
C24—N4—Ru1	116.9 (4)	C23—C22—H22	120.7
C29—N5—C25	118.7 (5)	C22—C23—C24	120.3 (6)
C29—N5—Ru1	126.2 (4)	C22—C23—H23	119.8
C25—N5—Ru1	114.8 (4)	C24—C23—H23	119.8
N1—C1—C2	123.2 (6)	N4—C24—C23	120.6 (6)
N1—C1—H1	118.4	N4—C24—C25	114.9 (5)
C2—C1—H1	118.4	C23—C24—C25	124.6 (5)
C1—C2—C3	119.0 (6)	N5—C25—C26	121.5 (6)
C1—C2—H2	120.5	N5—C25—C24	114.6 (5)
C3—C2—H2	120.5	C26—C25—C24	123.9 (6)
C2—C3—C4	119.4 (6)	C27—C26—C25	118.4 (6)
C2—C3—H3	120.3	C27—C26—H26	120.8
C4—C3—H3	120.3	C25—C26—H26	120.8
C3—C4—C5	119.0 (6)	C28—C27—C26	120.1 (6)
C3—C4—H4	120.5	C28—C27—H27	119.9
C5—C4—H4	120.5	C26—C27—H27	119.9
N1—C5—C4	121.7 (5)	C29—C28—C27	118.7 (6)
N1—C5—C6	114.8 (5)	C29—C28—H28	120.6
C4—C5—C6	123.5 (5)	C27—C28—H28	120.6
N2—C6—C7	120.3 (5)	N5—C29—C28	122.5 (6)
N2—C6—C5	112.7 (5)	N5—C29—H29	118.8
C7—C6—C5	127.0 (5)	C28—C29—H29	118.8
C6—C7—C8	118.5 (6)	F1—P2—F5	91.4 (5)
C6—C7—H7	120.7	F1—P2—F2	92.0 (5)
C8—C7—H7	120.7	F5—P2—F2	91.8 (5)
C9—C8—C7	120.4 (5)	F1—P2—F6	90.3 (5)
C9—C8—P1	122.5 (5)	F5—P2—F6	176.3 (6)
C7—C8—P1	117.1 (5)	F2—P2—F6	91.4 (5)
C8—C9—C10	119.6 (6)	F1—P2—F3	177.0 (7)
C8—C9—H9	120.2	F5—P2—F3	90.5 (6)
C10—C9—H9	120.2	F2—P2—F3	90.3 (6)
N2—C10—C9	119.4 (5)	F6—P2—F3	87.7 (5)
N2—C10—C11	113.2 (5)	F1—P2—F4	88.0 (5)
C9—C10—C11	127.4 (5)	F5—P2—F4	88.1 (4)
C12—C11—N3	121.4 (5)	F2—P2—F4	179.9 (7)
C12—C11—C10	124.3 (5)	F6—P2—F4	88.7 (4)
N3—C11—C10	114.2 (5)	F3—P2—F4	89.7 (5)
C11—C12—C13	119.7 (6)	F2B—P2B—F1B	90.1 (10)
C11—C12—H12	120.1	F2B—P2B—F5B	90.6 (11)
C13—C12—H12	120.1	F1B—P2B—F5B	90.1 (10)
C12—C13—C14	119.5 (6)	F2B—P2B—F3B	89.1 (10)
C12—C13—H13	120.3	F1B—P2B—F3B	178.5 (13)
C14—C13—H13	120.3	F5B—P2B—F3B	91.3 (11)
C15—C14—C13	118.5 (6)	F2B—P2B—F4B	178.7 (14)
C15—C14—H14	120.7	F1B—P2B—F4B	91.1 (11)
C13—C14—H14	120.7	F5B—P2B—F4B	89.1 (11)
N3—C15—C14	123.4 (6)	F3B—P2B—F4B	89.7 (11)
N3—C15—H15	118.3	F2B—P2B—F6B	91.0 (11)
C14—C15—H15	118.3	F1B—P2B—F6B	89.3 (11)
C16—O2—P1	116.5 (5)	F5B—P2B—F6B	178.3 (14)
C17—C16—O2	109.0 (7)	F3B—P2B—F6B	89.4 (10)
C17—C16—H16A	109.9	F4B—P2B—F6B	89.3 (11)
O2—C16—H16A	109.9	N6—C30—C31	177.7 (14)
C17—C16—H16B	109.9	C30—C31—H31A	109.5
O2—C16—H16B	109.9	C30—C31—H31B	109.5
H16A—C16—H16B	108.3	H31A—C31—H31B	109.5
C16—C17—H17A	109.5	C30—C31—H31C	109.5
C16—C17—H17B	109.5	H31A—C31—H31C	109.5
H17A—C17—H17B	109.5	H31B—C31—H31C	109.5
C16—C17—H17C	109.5	H4A—O4—H4B	112.0 (2)
H17A—C17—H17C	109.5	H5A—O5—H5B	112.0 (2)
			
C5—N1—C1—C2	1.0 (8)	C10—C11—C12—C13	−175.1 (6)
Ru1—N1—C1—C2	−174.8 (4)	C11—C12—C13—C14	−1.3 (9)
N1—C1—C2—C3	−1.7 (9)	C12—C13—C14—C15	−0.3 (9)
C1—C2—C3—C4	1.0 (9)	C11—N3—C15—C14	0.2 (8)
C2—C3—C4—C5	0.5 (9)	Ru1—N3—C15—C14	174.8 (4)
C1—N1—C5—C4	0.6 (8)	C13—C14—C15—N3	0.9 (9)
Ru1—N1—C5—C4	177.0 (4)	O1—P1—O2—C16	11.7 (6)
C1—N1—C5—C6	−177.6 (5)	O3B—P1—O2—C16	137.0 (6)
Ru1—N1—C5—C6	−1.2 (6)	O3—P1—O2—C16	148.6 (12)
C3—C4—C5—N1	−1.3 (9)	C8—P1—O2—C16	−112.2 (5)
C3—C4—C5—C6	176.7 (5)	P1—O2—C16—C17	−90.4 (8)
C10—N2—C6—C7	1.1 (8)	O1—P1—O3—C18	67 (4)
Ru1—N2—C6—C7	−178.8 (4)	O2—P1—O3—C18	−58 (3)
C10—N2—C6—C5	179.4 (5)	O3B—P1—O3—C18	85 (5)
Ru1—N2—C6—C5	−0.5 (6)	C8—P1—O3—C18	−166 (3)
N1—C5—C6—N2	1.1 (7)	P1—O3—C18—C19	−93 (4)
C4—C5—C6—N2	−177.0 (5)	O1—P1—O3B—C18B	62.8 (8)
N1—C5—C6—C7	179.3 (6)	O2—P1—O3B—C18B	−62.9 (8)
C4—C5—C6—C7	1.2 (9)	O3—P1—O3B—C18B	−102 (4)
N2—C6—C7—C8	−1.9 (9)	C8—P1—O3B—C18B	−176.3 (7)
C5—C6—C7—C8	−179.9 (5)	P1—O3B—C18B—C19B	166.6 (9)
C6—C7—C8—C9	0.4 (9)	C24—N4—C20—C21	−1.4 (9)
C6—C7—C8—P1	178.7 (4)	Ru1—N4—C20—C21	175.1 (5)
O1—P1—C8—C9	156.3 (5)	N4—C20—C21—C22	1.0 (10)
O2—P1—C8—C9	−79.0 (6)	C20—C21—C22—C23	0.3 (11)
O3B—P1—C8—C9	35.0 (6)	C21—C22—C23—C24	−1.1 (12)
O3—P1—C8—C9	17.0 (14)	C20—N4—C24—C23	0.6 (9)
O1—P1—C8—C7	−21.9 (6)	Ru1—N4—C24—C23	−176.2 (5)
O2—P1—C8—C7	102.8 (5)	C20—N4—C24—C25	−177.8 (5)
O3B—P1—C8—C7	−143.2 (5)	Ru1—N4—C24—C25	5.3 (7)
O3—P1—C8—C7	−161.1 (14)	C22—C23—C24—N4	0.6 (11)
C7—C8—C9—C10	1.7 (9)	C22—C23—C24—C25	178.9 (7)
P1—C8—C9—C10	−176.4 (4)	C29—N5—C25—C26	0.5 (9)
C6—N2—C10—C9	1.0 (8)	Ru1—N5—C25—C26	−174.6 (5)
Ru1—N2—C10—C9	−179.0 (4)	C29—N5—C25—C24	−179.5 (5)
C6—N2—C10—C11	−176.9 (5)	Ru1—N5—C25—C24	5.4 (6)
Ru1—N2—C10—C11	3.0 (6)	N4—C24—C25—N5	−7.0 (8)
C8—C9—C10—N2	−2.4 (8)	C23—C24—C25—N5	174.6 (6)
C8—C9—C10—C11	175.2 (5)	N4—C24—C25—C26	172.9 (6)
C15—N3—C11—C12	−1.8 (8)	C23—C24—C25—C26	−5.4 (10)
Ru1—N3—C11—C12	−177.2 (4)	N5—C25—C26—C27	0.1 (10)
C15—N3—C11—C10	175.9 (5)	C24—C25—C26—C27	−179.9 (6)
Ru1—N3—C11—C10	0.5 (6)	C25—C26—C27—C28	0.0 (10)
N2—C10—C11—C12	175.5 (5)	C26—C27—C28—C29	−0.5 (10)
C9—C10—C11—C12	−2.3 (9)	C25—N5—C29—C28	−1.1 (9)
N2—C10—C11—N3	−2.2 (7)	Ru1—N5—C29—C28	173.4 (5)
C9—C10—C11—N3	−179.9 (5)	C27—C28—C29—N5	1.2 (10)
N3—C11—C12—C13	2.4 (9)		
